# Supplementary material for: Drug Inhibition of Redox Factor-1 Restores Hypoxia-Driven Changes in Tuberous Sclerosis Complex 2 Deficient Cells
Source: Cancers (Basel). 2022 Dec 15;14(24):6195. doi: 10.3390/cancers14246195 (PMC9776744; doi:10.3390/cancers14246195)

Figure 1A

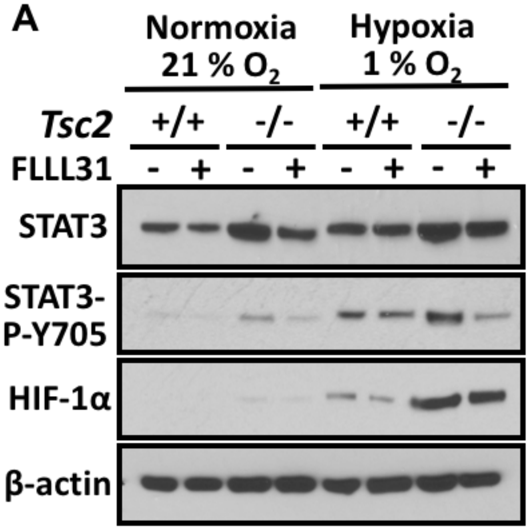

STAT3

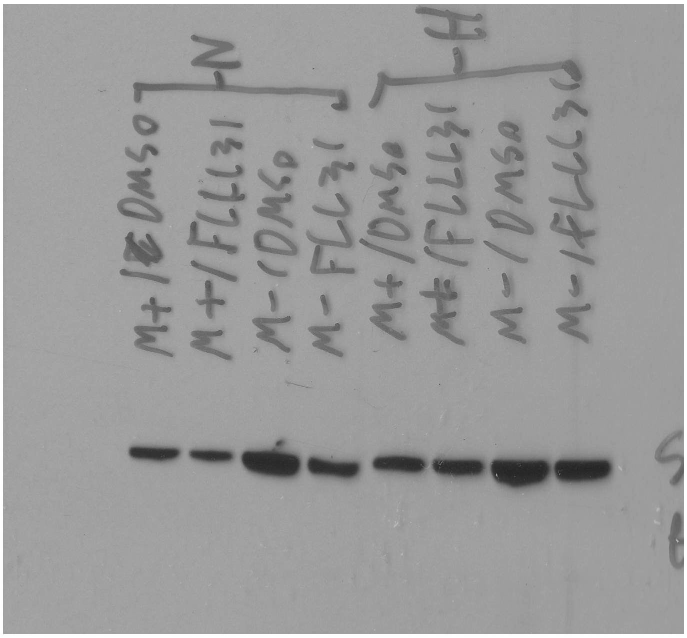

(P-Y705)-STAT3

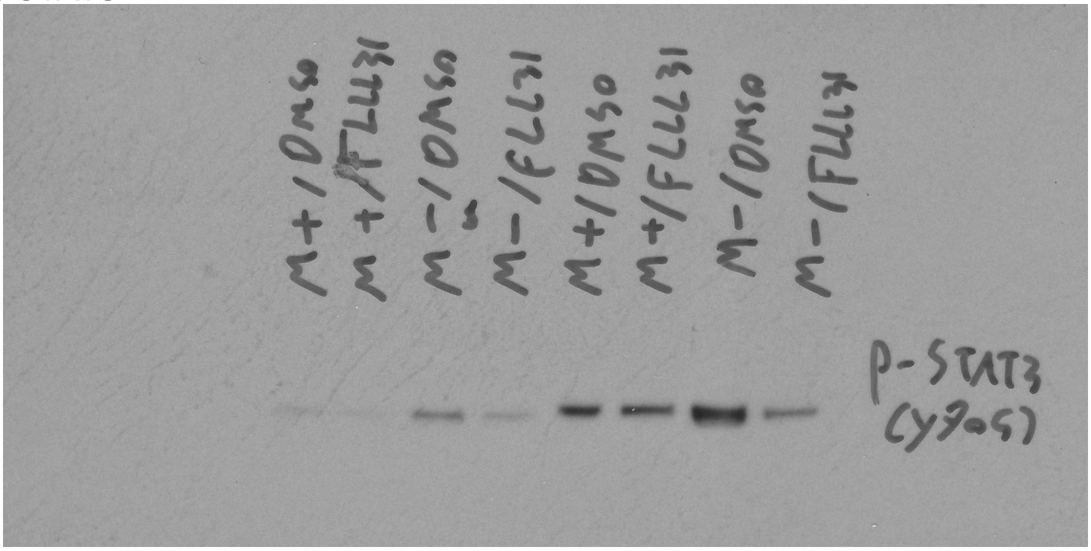

HIF-1α

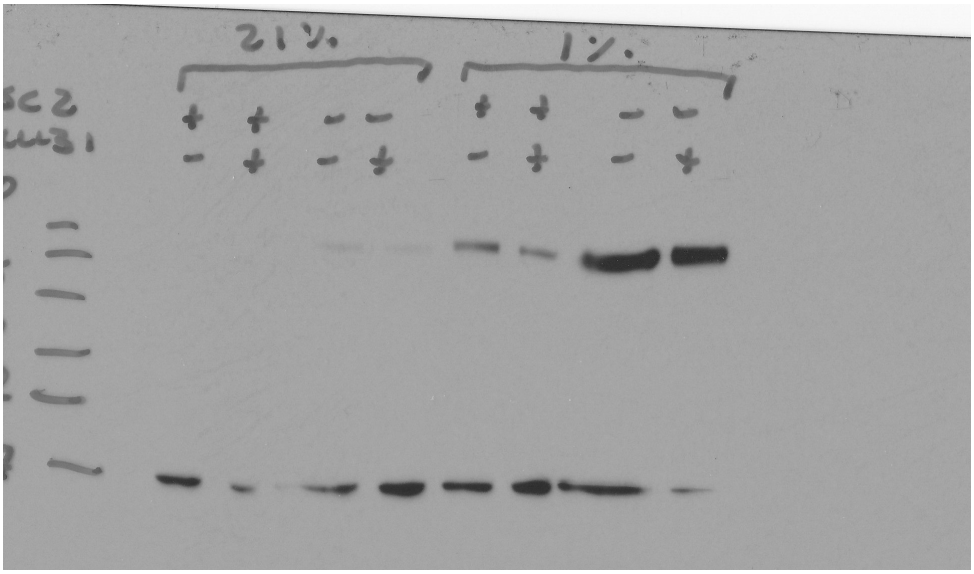

β-actin

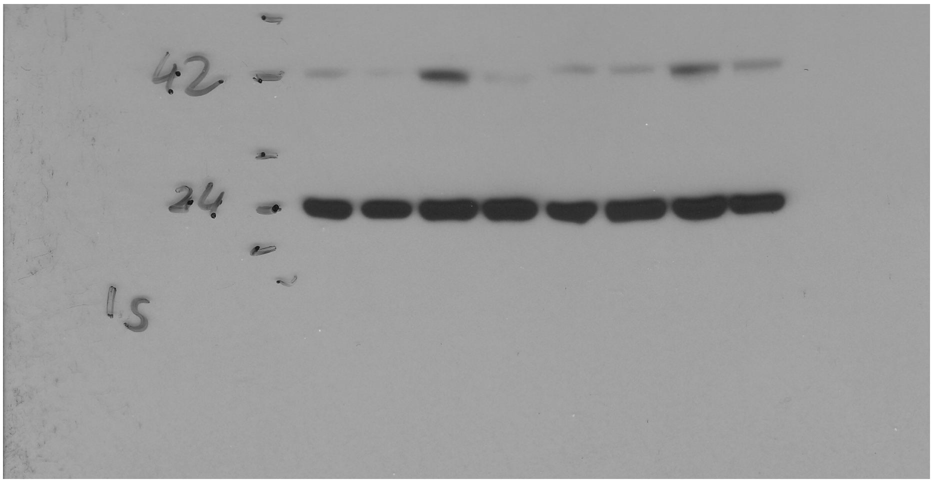

Figure 1E

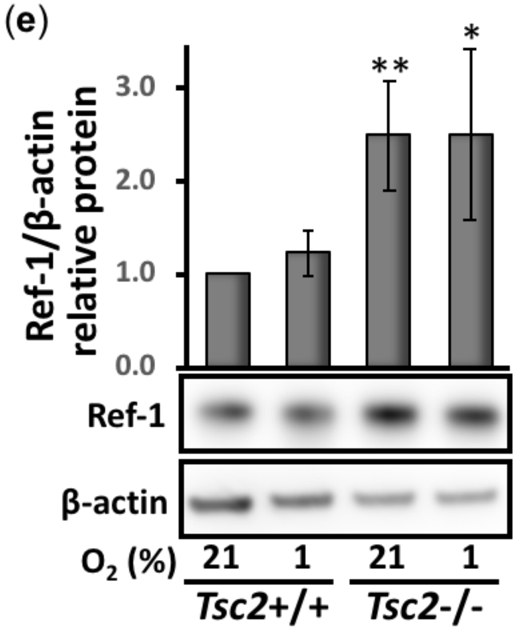

(e)
Ref-1/β-actin relative protein
3.0
2.0
1.0
0.0
**
*
Ref-1
β-actin
O2 (%)
21
1
21
1
Tsc2+/+
Tsc2-/-

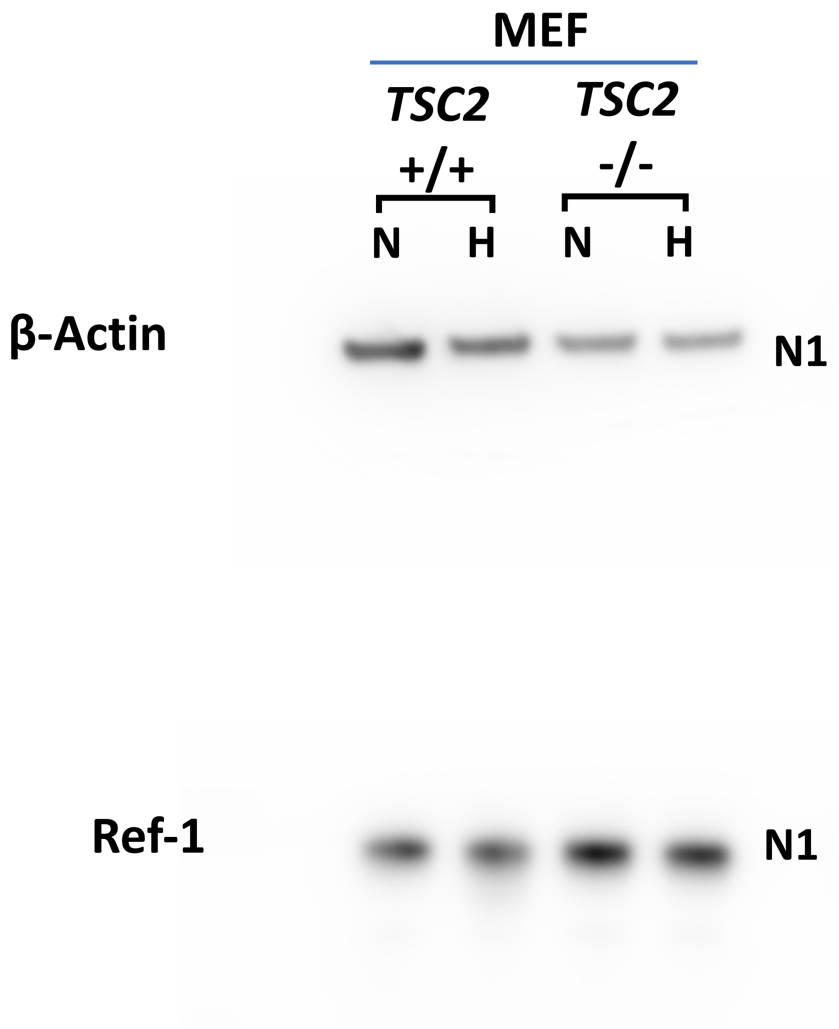

MEF
TSC2 +/+
TSC2 -/-
N
H
N
H
β-Actin
N1
Ref-1
N1

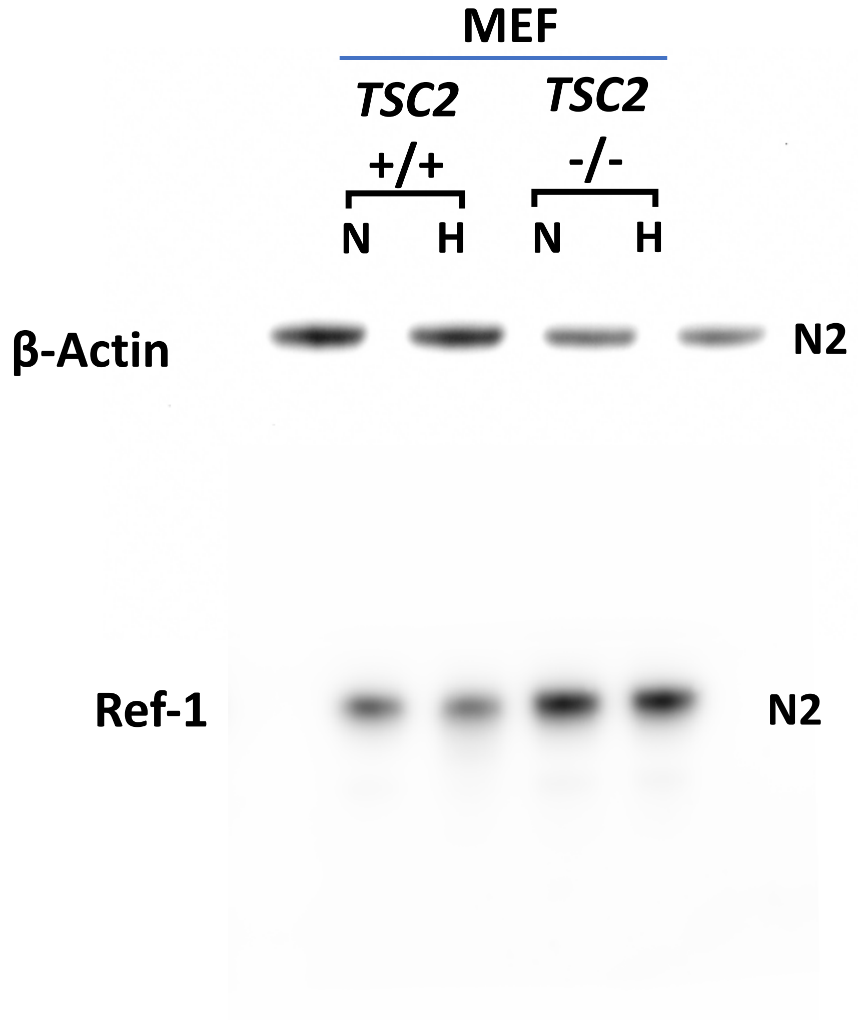
MEF
TSC2 +/+
TSC2 -/-
N H N H
β-Actin
N2
Ref-1
N2

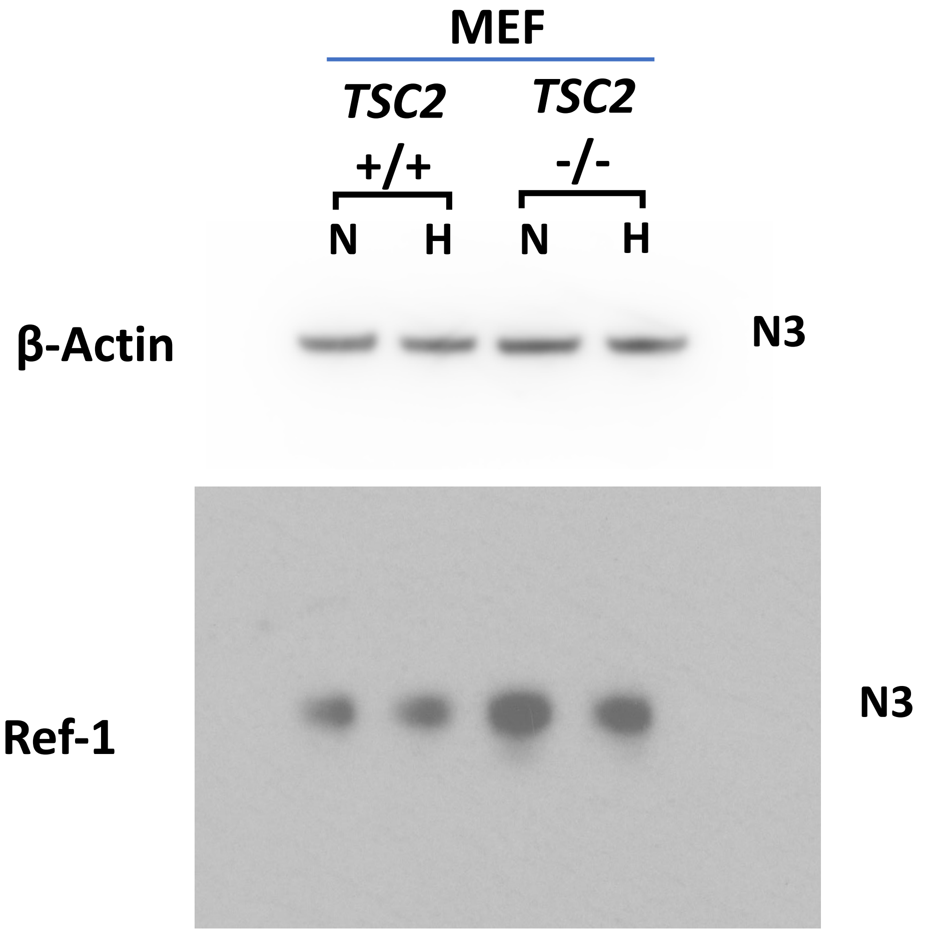
MEF
TSC2 +/+
TSC2 -/-
N H N H
β-Actin
N3
Ref-1
N3

Figure 2D

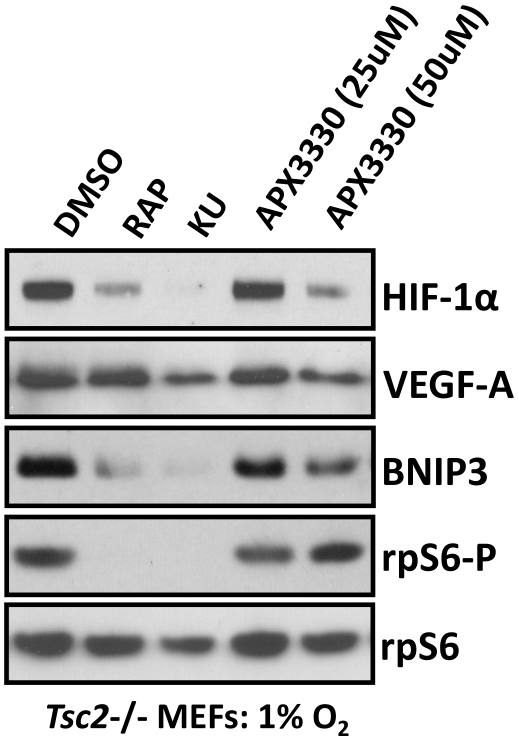

HIF-1α

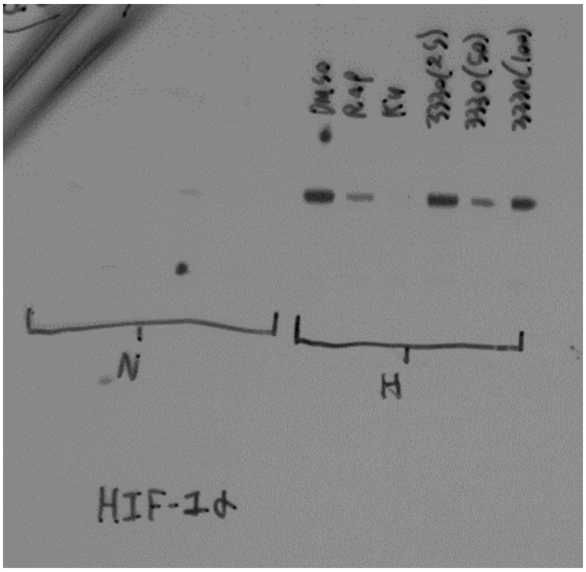

VEGF-A

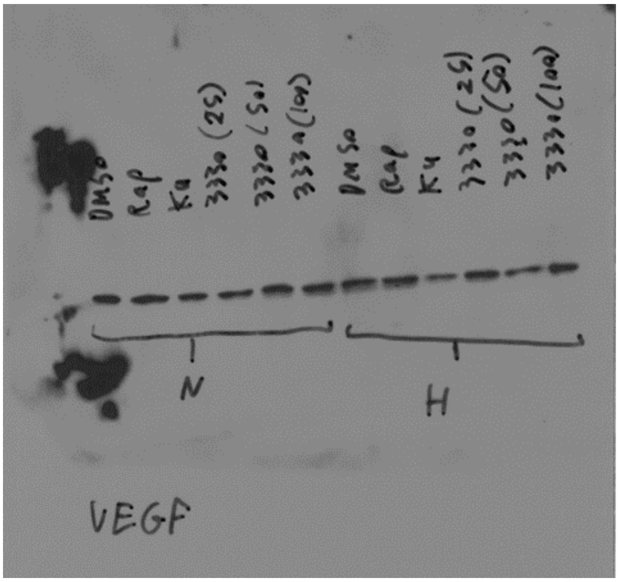

BNIP3

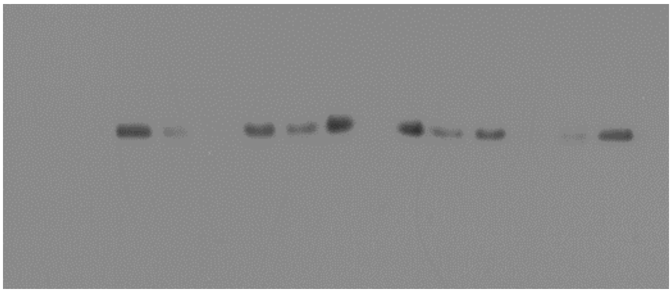

rpS6-P

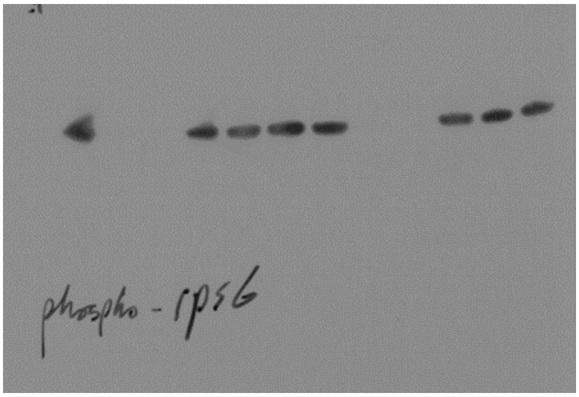

rpS6

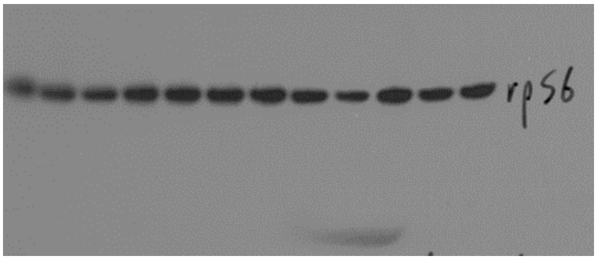

Figure 5

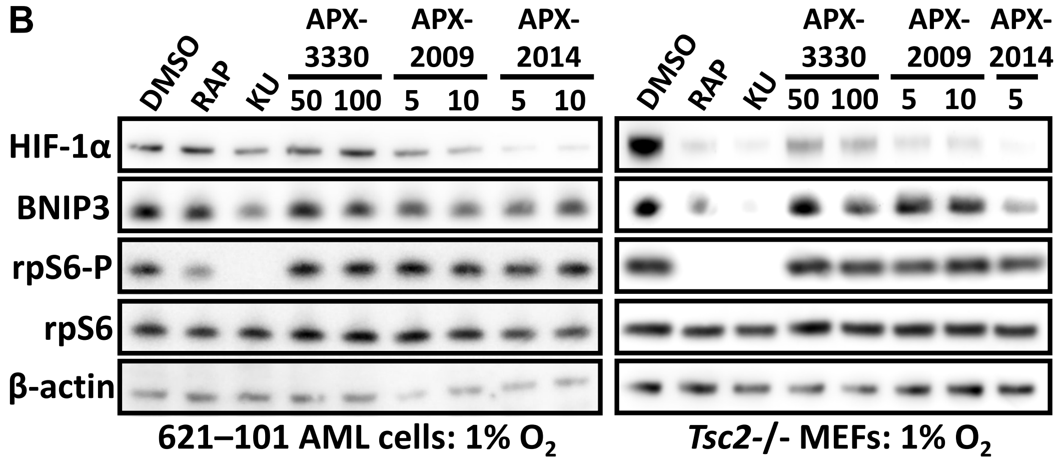

621–101 AML cells: 1% O_2

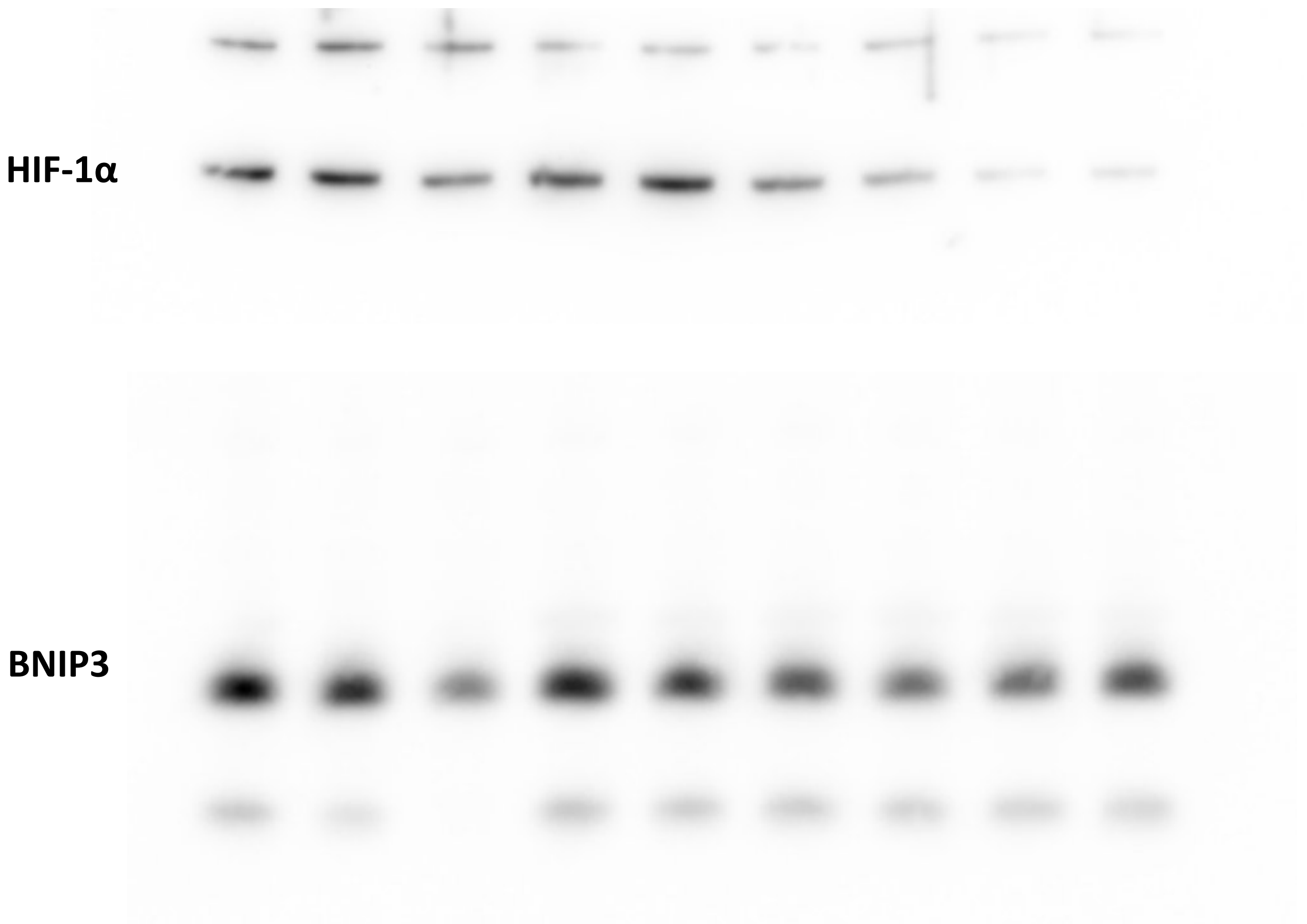

621–101 AML cells: 1% O_2

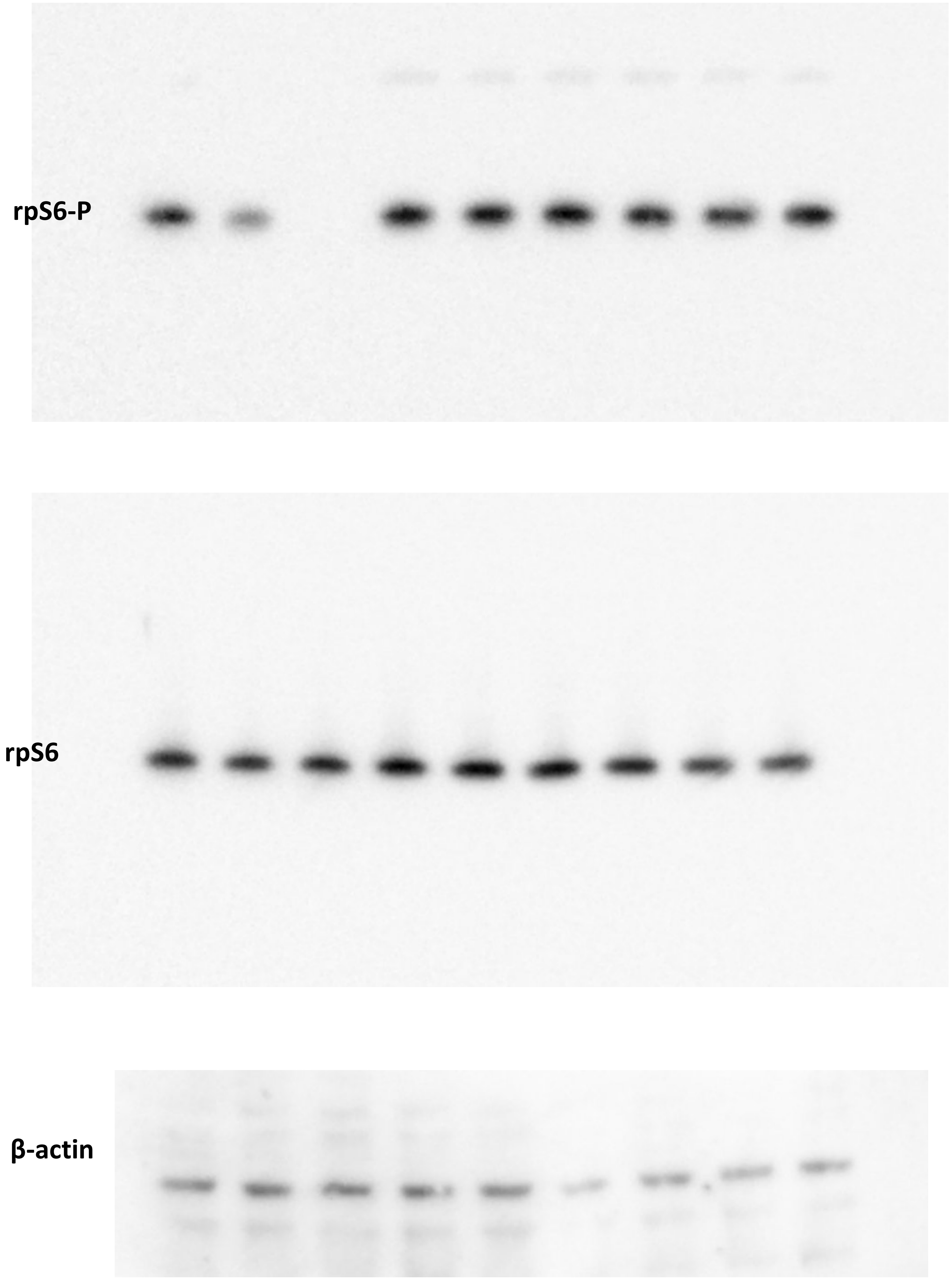

Tsc2-/- MEFs: 1% O_2

HIF-1α

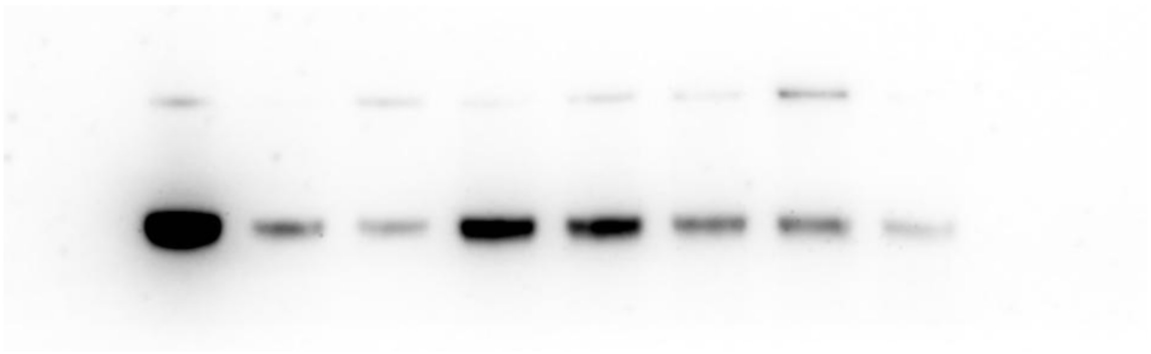

BNIP3

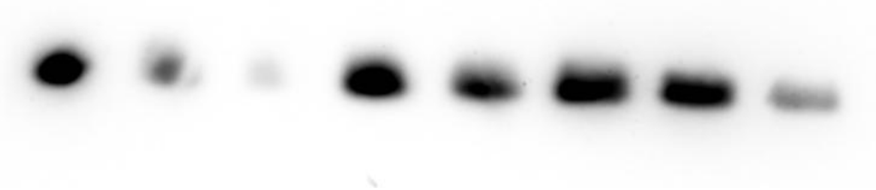

Tsc2-/- MEFs: 1% O_2

rpS6-P

rpS6

β-actin

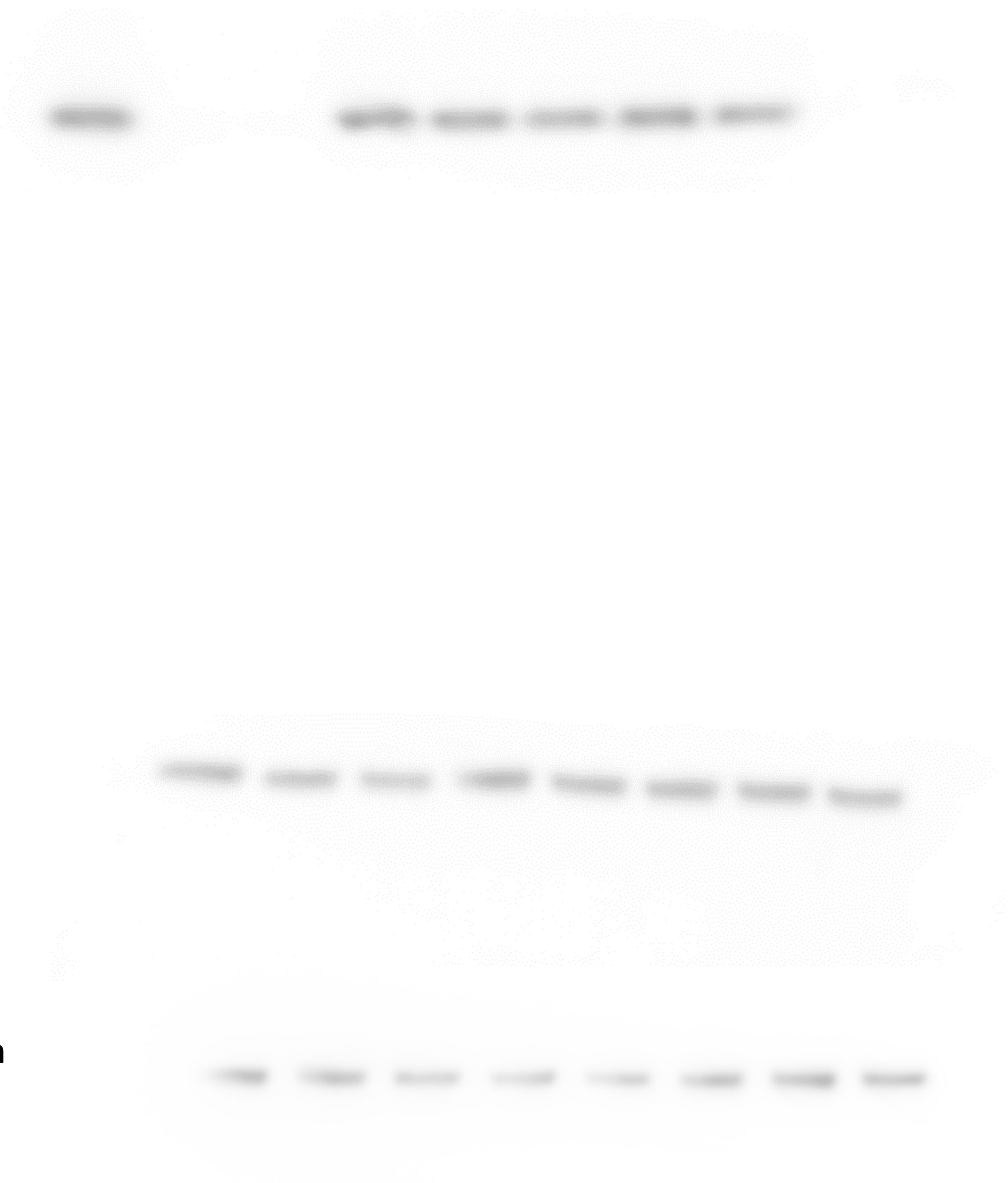

Supplementary Figure 1

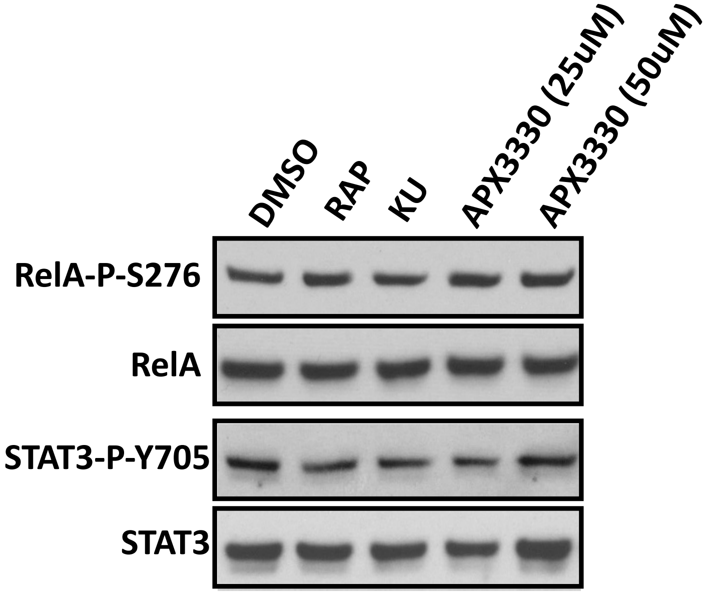

RelA-P-S276

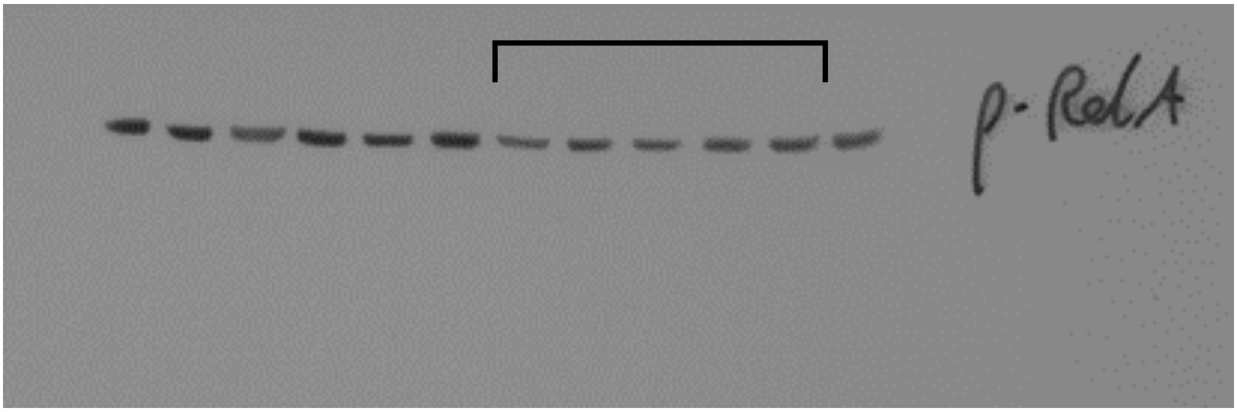

RelA

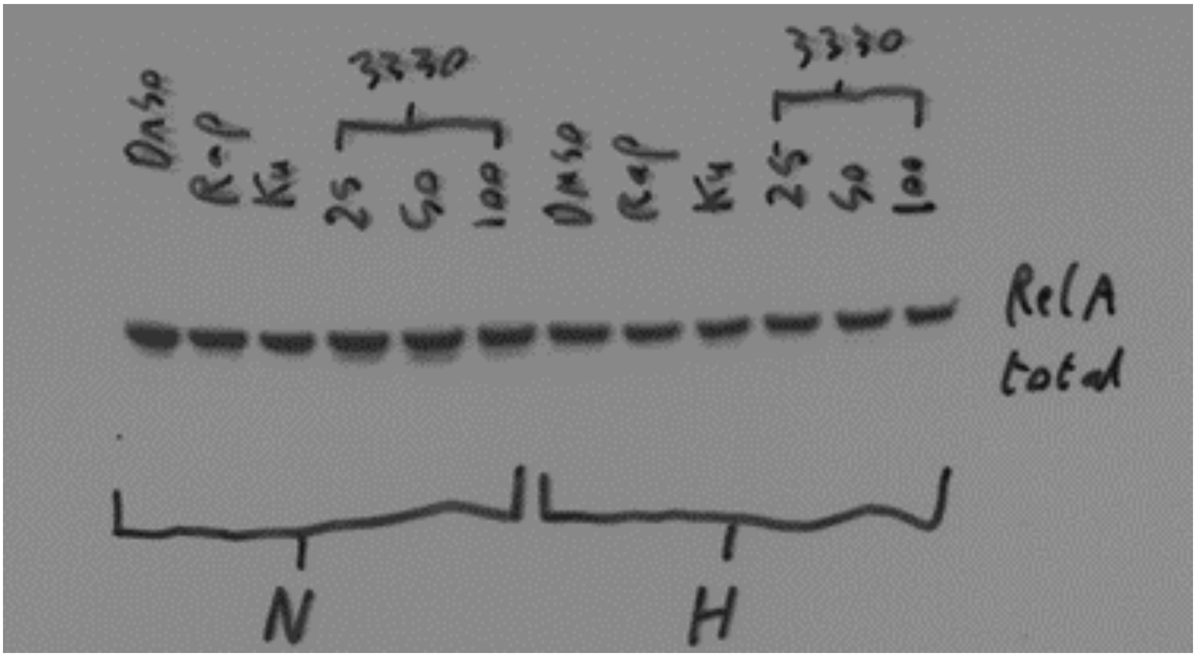

STAT3-P-Y705

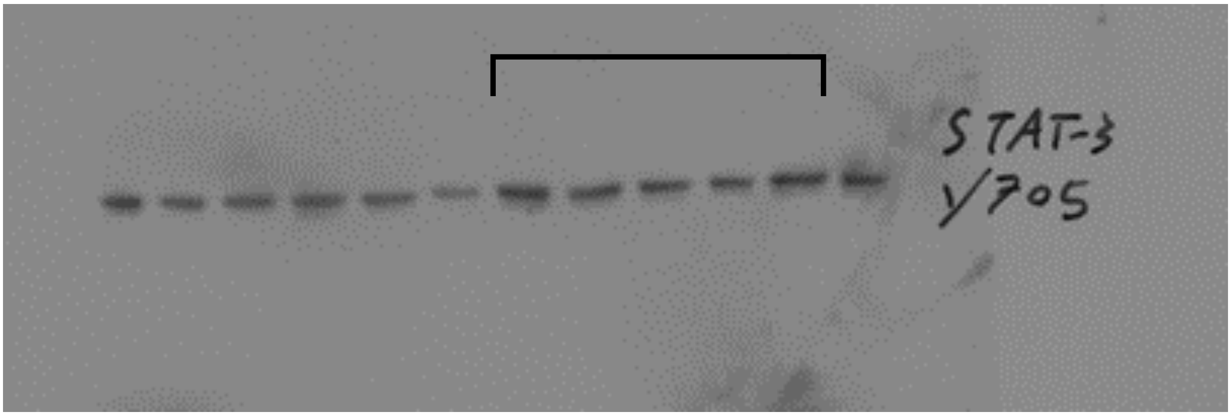

STAT3

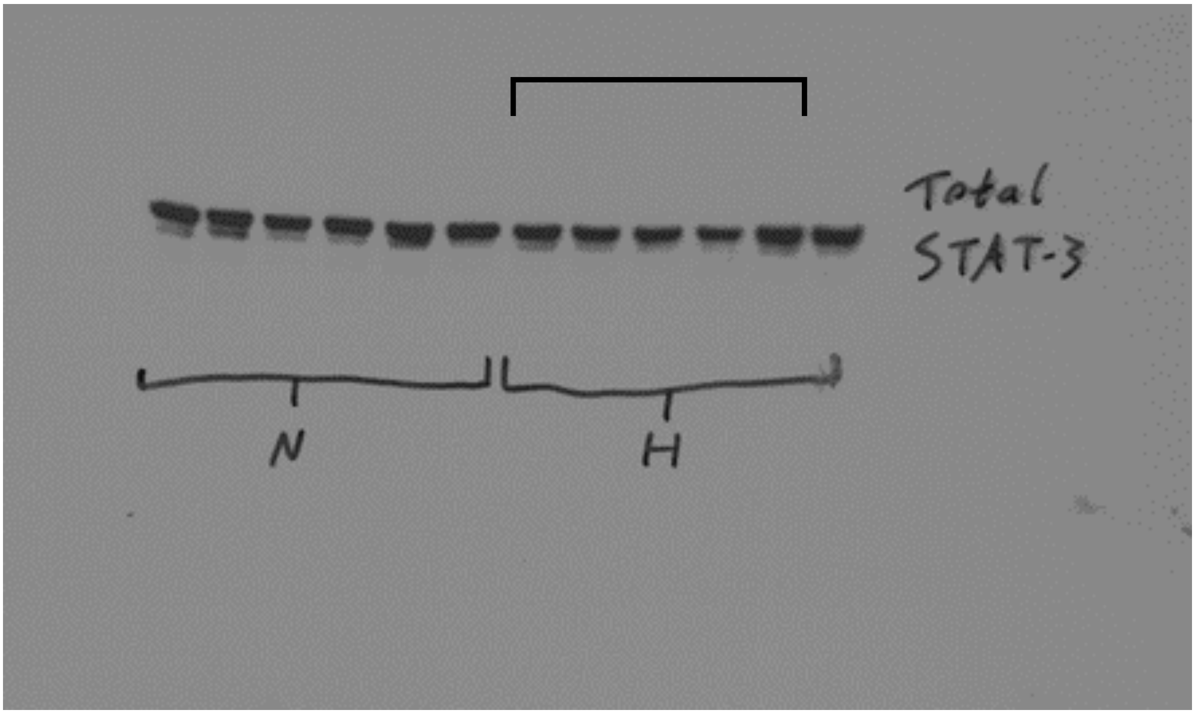

Supplement: Supplementary file 1 [file cancers-14-06195-s001.zip › cancers-1933841.pdf]
