# Supplementary material for: Drug Inhibition of Redox Factor-1 Restores Hypoxia-Driven Changes in Tuberous Sclerosis Complex 2 Deficient Cells
Source: Cancers (Basel). 2022 Dec 15;14(24):6195. doi: 10.3390/cancers14246195 (PMC9776744; doi:10.3390/cancers14246195)
Supplement: Supplementary file 1 [file cancers-14-06195-s001.zip › Supplementary Figure S1.pdf]

cancers

MDPI

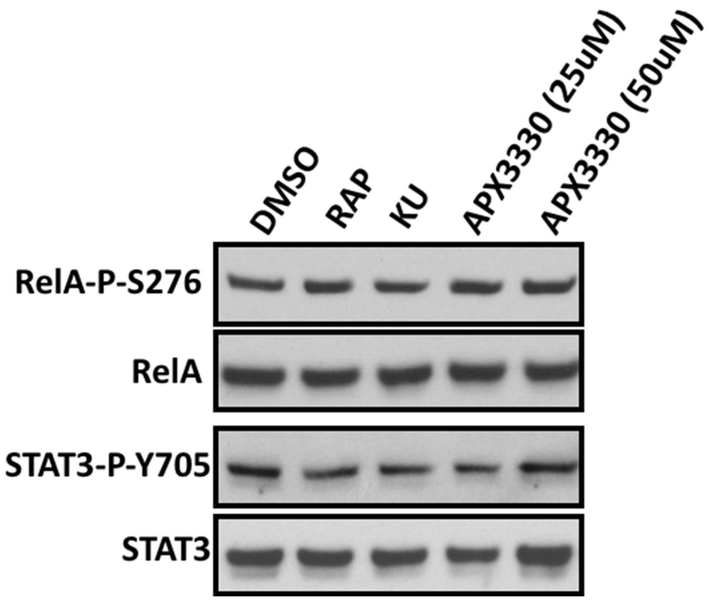

Supplementary Figure S1. APX3330 and mTOR inhibitors do not reduce RelA S276 or STAT3 Y705 phosphorylation in *Tsc2*−/− MEFs. *Tsc2*−/− MEFs were treated with either DMSO, 50 nM rapamycin, 1 μM Ku-0063794 (KU) or APX3330 (25 or 50 μM) for 18 h under hypoxia (1 % O_2). Western blots for total and phosphorylated RelA and STAT3 were carried out, *n*=3.
