# Supplementary material for: Drug Inhibition of Redox Factor-1 Restores Hypoxia-Driven Changes in Tuberous Sclerosis Complex 2 Deficient Cells
Source: Cancers (Basel). 2022 Dec 15;14(24):6195. doi: 10.3390/cancers14246195 (PMC9776744; doi:10.3390/cancers14246195)
Supplement: Supplementary file 1 [file cancers-14-06195-s001.zip › Supplementary Figure S2.pdf]

cancers

MDPI

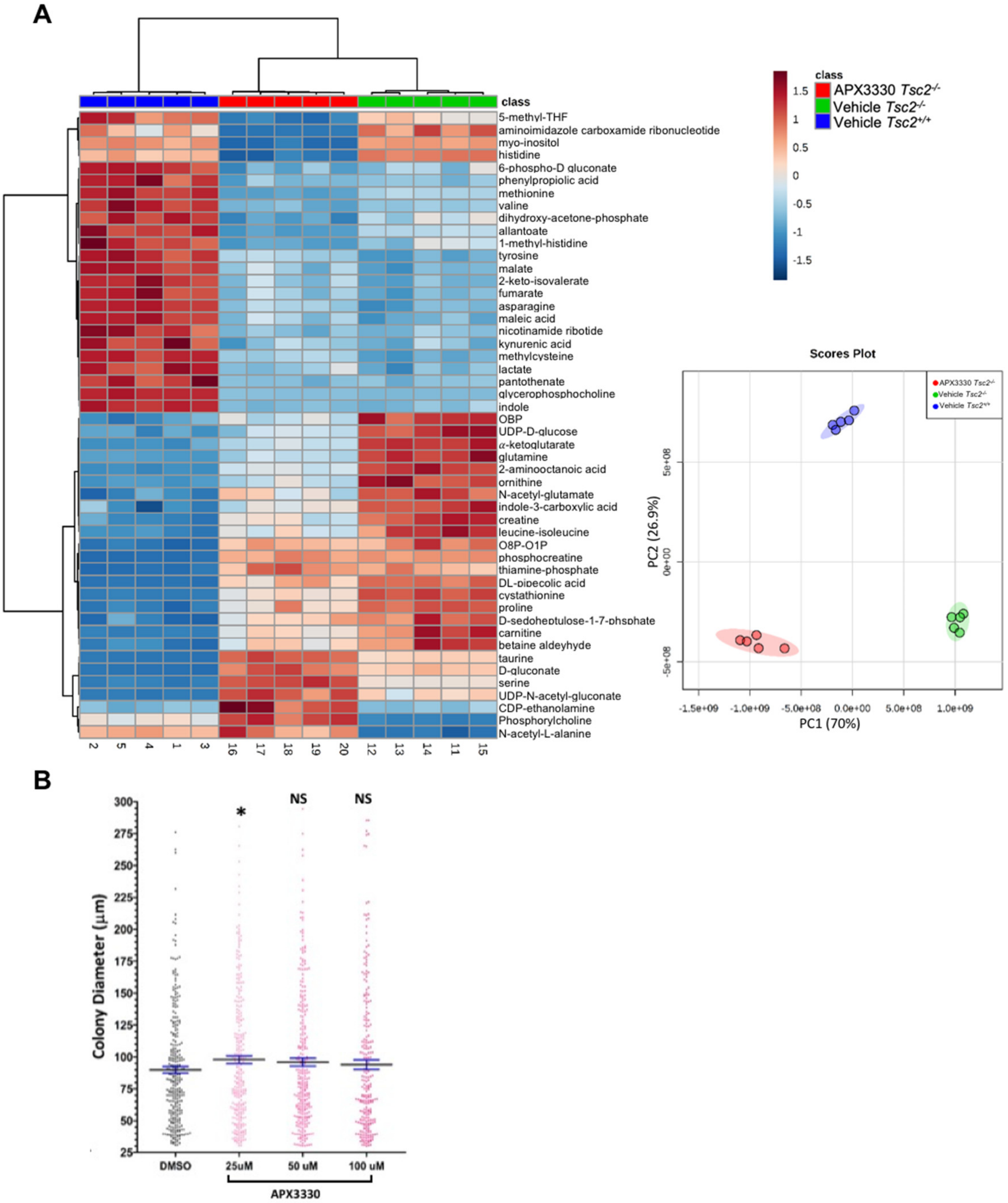

Supplementary Figure S2. (**A**) Heatmap of metabolite data on *Tsc2*+/+ and *Tsc2*−/− MEFs treated with vehicle only (DMSO) or 25 µM APX3330 (over-night treatment, 18 h). (**B**) Tumor formation assay of 621-102 AML cells in 15 % (v/v) FBS with either DMSO or APX3330 (25, 50 and 100 µM) and scored as colony diameter (µm). * $p<0.05$ and not-significant (NS).
