# Supplementary material for: Drug Inhibition of Redox Factor-1 Restores Hypoxia-Driven Changes in Tuberous Sclerosis Complex 2 Deficient Cells
Source: Cancers (Basel). 2022 Dec 15;14(24):6195. doi: 10.3390/cancers14246195 (PMC9776744; doi:10.3390/cancers14246195)
Supplement: Supplementary file 1 [file cancers-14-06195-s001.zip › Supplementary Figure S3.pdf]

cancers

MDPI

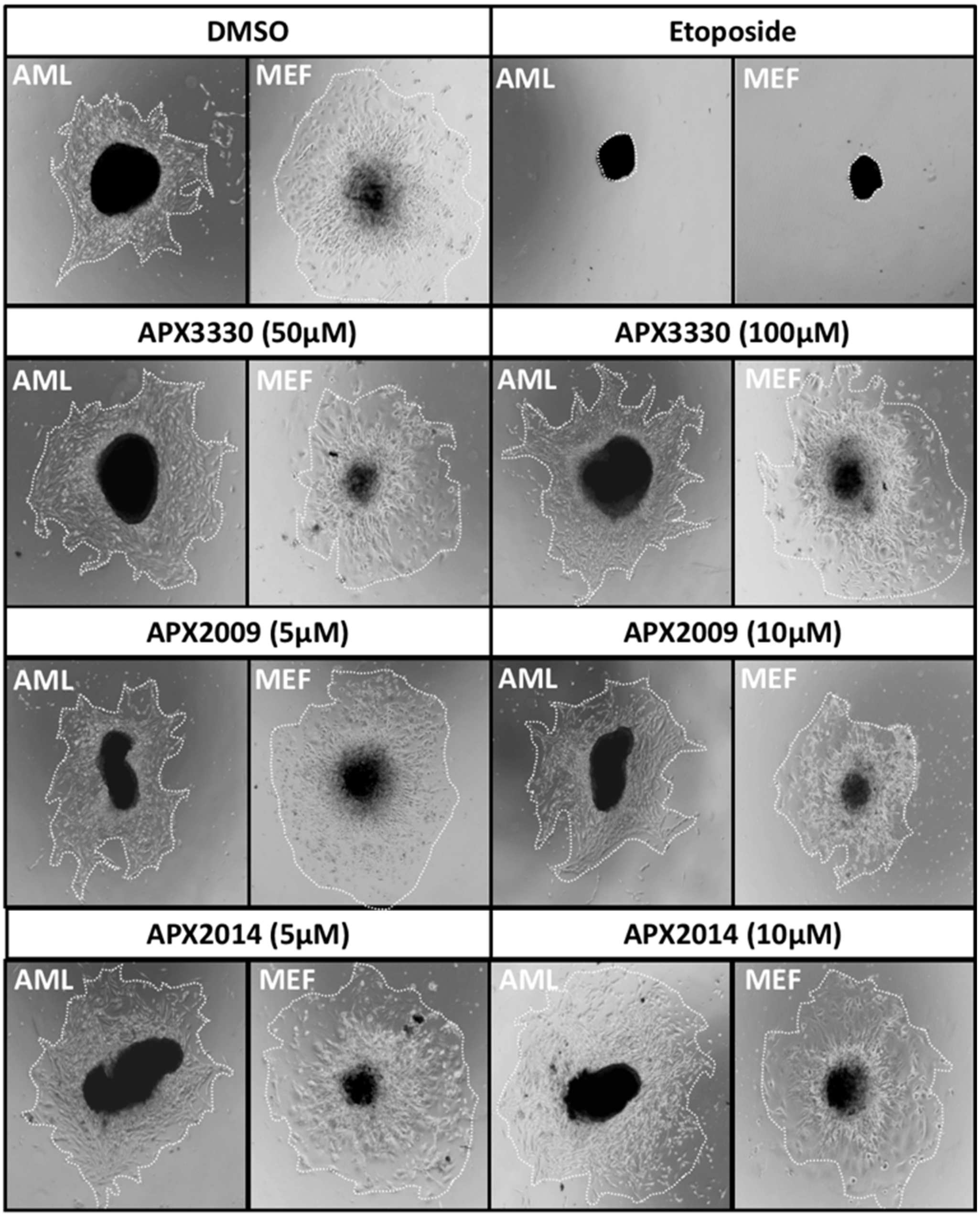

Supplementary Figure S3. *Tsc2*−/− MEFs and 621-102 AML tumor spheroids recover after Ref-1 inhibitor treatment. *Tsc2*−/− MEFs and 621-102 AML cells were plated on soft agar coated 96 well plates and treated with either vehicle only (DMSO), APX3330 (50 or 100 µM), APX2009 (5 or 10 µM), APX2014 (5 or 10 µM) or 10 µM etoposide for 11 days. Spheroids were then re-plated onto standard tissue culture plates and grown under drug-free conditions. Images were taken after 48 h. Relative outgrowth areas are outlined.
